# Association of interstage timing and prior surgical response with outcomes following staged bilateral total knee arthroplasty: A retrospective cohort study

**DOI:** 10.1002/jeo2.70809

**Published:** 2026-06-23

**Authors:** Jake M. Laverdiere, Ekrem M. Ayhan, Thomas P. Giannasca, Jordan A. Bauer, Baseel M. Hamideh, Martinus Megalla, Matthew J. Grosso

**Affiliations:** ^1^ Connecticut Joint Replacement Institute at Saint Francis Hospital Hartford Connecticut USA; ^2^ Frank H. Netter, School of Medicine, North Haven Connecticut Joint Replacement Institute at Saint Francis Hospital Hartford Connecticut USA; ^3^ Department of Orthopaedic Surgery University of Connecticut Health Center Farmington Connecticut USA

**Keywords:** complications, minimal clinically important difference, patient‐reported outcomes, surgical timing, total knee arthroplasty

## Abstract

**Purpose:**

Staged bilateral total knee arthroplasty (BTKA) is increasingly performed, yet the influence of inter‐stage timing on complications and patient‐reported outcomes (PROs) remains unclear. This study evaluated whether timing between staged BTKA procedures was independently associated with 90‐day complications or failure to achieve the minimal clinically important difference (MCID) in 1‐year PROs after the second surgery.

**Methods:**

Retrospective study of 2410 patients who underwent staged BTKA at a high‐volume single institution (2014–2024). Timing was assessed using quartiles, a ≤6 versus >6‐week cutoff, and as a continuous variable. The primary outcome was any 90‐day complication after the second procedure, as defined by The Knee Society Guidelines. Secondary outcomes included failure to achieve MCID at 1‐year for the knee injury and osteoarthritis outcome score for joint replacement (KOOS‐JR) and veterans RAND 12‐item survey (VRS‐12) physical (PCS) and mental component scores (MCS). Multivariable regression adjusted for demographics, second‐stage preoperative PROs, and complications after the first stage.

**Results:**

Median time between stages was 245 days. Timing was not independently associated with 90‐day complications or failure to achieve MCID at 1‐year in adjusted models. Prior complications after the first procedure (odds ratio [OR]: 4.01, 95% confidence interval [CI]: 2.21–7.26; *p* < 0.001) and ASA III–IV (OR: 1.60, 95% CI: 1.05–2.43; *p* = 0.028) were associated with increased complication risk. Failure to achieve MCID after the first surgery was strongly associated with subsequent MCID failure following the second surgery (KOOS‐JR: OR: 26.34; VRS‐12 PCS: OR: 31.67; MCS: OR: 8.69; all *p* < 0.001).

**Conclusions:**

Inter‐stage timing was not independently associated with complications or failure to achieve MCID at 1‐year after adjustment for patient‐level factors. Prior surgical response and ASA classification demonstrated stronger associations with postoperative outcomes following the second stage than timing alone. These findings support individualised surgical planning and shared decision‐making rather than reliance on fixed interval thresholds.

**Level of Evidence:**

Level III.

**Abbreviations**
AKI: acute kidney injury
ANOVA: analysis of variance
ASA: American Society of Anesthesiologists
BMI: body mass index
BTKA: bilateral total knee arthroplasty
CCI: Charlson Comorbidity Index
CI: confidence interval
CVA: cerebrovascular accident
ED: emergency department
KOOS‐JR: knee injury and osteoarthritis outcome score for joint replacement
MCID: minimal clinically important difference
MI: myocardial infarction
OR: odds ratio
PJI: periprosthetic joint infection
PNA: pneumonia
PRO: patient‐reported outcome
SSI: surgical site infection
TIA: transient ischaemic attack
TKA: total knee arthroplasty
VIF: variance inflation factors
VRS‐12 MCS: veterans RAND 12‐item survey mental component score
VRS‐12 PCS: veterans RAND 12‐item survey physical component score
VTE: venous thromboembolism

## INTRODUCTION

Total knee arthroplasty (TKA) is one of the most common and effective surgical interventions for end‐stage osteoarthritis [29]. With advancements in arthroplasty techniques, an ageing population, and the high prevalence of bilateral disease, staged bilateral total knee arthroplasty (BTKA) has become increasingly common. Bilateral disease is associated with greater pain, stiffness, and functional impairment compared to unilateral pathology, often prompting patients to pursue BTKA [13, 18, 26, 33].

Simultaneous BTKA has become less common compared to staged BTKA, partly due to evidence for increased complication risk in this simultaneous population [11, 23]. For those who ultimately receive BTKA in stages, the optimal timing between procedures remains unclear. Some studies suggest that early second‐stage surgery may expedite recovery and improve satisfaction without added risk [2, 6], while others report increased complications when the interval is less than 6 weeks [1, 24]. Conversely, several investigations have found no association between timing and complications or poor functional outcomes [4, 7, 14, 20, 21]. While these studies contribute valuable insight, they are constrained by small sample sizes or lack of adjustment for key clinical variables such as prior complications and comorbidity burden. In addition, few studies have modelled time as a continuous variable or examined the effect of inter‐stage timing on patient‐reported outcomes (PROs).

Given the physical and logistical demands of postoperative recovery, it is essential to determine whether inter‐stage timing meaningfully influences complication rates and PROs. Clarifying this relationship is central to shared decision‐making, enabling patients and surgeons to align surgical timing with medical risk, recovery goals, and life demands. The aim of this study was to evaluate whether time between BTKA stages influences 90‐day complications or PROs after the second surgery. We hypothesised that timing would not significantly impact complications or PROs after accounting for patient‐level risk factors.

## METHODS

### Study design and inclusion criteria

This was a retrospective cohort study of patients ages 18–89 who underwent elective TKA and a subsequent contralateral TKA between the dates of January 1, 2014, and January 31, 2024, using an institutional database from a high‐volume, single‐centre acute care hospital. The study was approved by the institutional review board prior to data collection. Patients with BTKA procedures performed simultaneously were excluded. Procedures performed for revision, fracture, or traumatic injury were also omitted.

Inter‐stage timing was modelled using three complementary approaches informed by prior literature and the distribution of the available dataset. Quartiles were used to evaluate potential nonlinear relationships across the full distribution of staging intervals. Timing was also assessed using a binary threshold (≤6 vs. >6 weeks) based on prior BTKA literature that identified 6 weeks as a clinically relevant staging interval associated with complication risk [1, 2, 3, 12]. Finally, timing was evaluated as a continuous variable scaled in 30‐day increments to assess for dose‐dependent associations.

Timing between procedures was likely determined through a shared decision‐making process between the surgeon and patient, rather than a standardised protocol. Factors influencing timing and surgical planning may have included symptom severity, recovery from the first stage, social support, logistical considerations, and surgeon discretion.

### Data collection and outcome measures

All data were extracted from electronic medical records. Demographic and clinical data for both the first and second procedure included age, body mass index (BMI), sex, smoking status, Charlson comorbidity index (CCI) and American Society of Anesthesiologists (ASA) classification. The primary outcome measure was pooled 90‐day complications after the second procedure. Complications were defined per the standardised list developed by The Knee Society [9]. These included instances of cerebrovascular accident (CVA), transient ischaemic attack (TIA), dislocations, periprosthetic fractures, tendon rupture, mechanical failure, implant malposition, venous thromboembolism (VTE), surgical wound dehiscence, surgical site infection (SSI), periprosthetic joint infection (PJI), nerve injury, haematoma, acute myocardial infarction (MI), cardiac arrest, cardiac arrhythmia, gastrointestinal haemorrhage, acute kidney injury (AKI), pneumonia (PNA), vascular injury, reoperation, emergency department (ED) visits, hospital readmissions, and mortality. Secondary outcomes included identifying predictors of failure to achieve the minimal clinically important difference (MCID) on PRO measures at 1‐year postoperatively. The PROs used included the knee injury and osteoarthritis outcomes score for joint replacement (KOOS‐JR) and the veterans RAND 12‐item (VRS‐12) physical (PCS) and mental component scores (MCS). Thresholds for MCID were derived from prior literature as a 16‐point increase for KOOS‐JR. scores [5] and a 5‐point increase for both VRS‐12 PCS and MCS [16, 30].

### Data analyses

A post‐hoc sensitivity analysis was conducted based on the number of patient charts that met inclusion criteria (*n* = 2410). Assuming approximately equal distribution across quartiles and an overall 90‐day complication rate of 4.7%, this sample size provided 80% power at *ɑ* = 0.05 to detect an absolute difference of ~4.0 percentage points in complication rates between quartiles. Distribution assumptions were verified statistically using the Shapiro–Wilk test. Normally distributed continuous data are presented as mean and standard deviation assessed using one‐way analysis of variance (ANOVA), Kruskal–Wallis test, independent samples *t*‐test, or Mann–Whitney *U* test. Categorical data are presented as frequencies and percentages which were compared using *Chi*‐square or Fisher's exact test when cell counts were <5. Bonferroni adjustment was applied to categorical variables with multiple comparisons. Demographics, surgical variables, 90‐day complications, and PROs for the second stage were assessed within both quartile and binary grouping modalities via univariable analysis. Multivariable logistic regression was performed to identify key predictors and assess the independent relationship of time on 90‐day complications and risk of failing to meet MCID for 1‐year PROs after the second surgery. Multivariable models assessed inter‐stage timing as quartiles, ≤6 vs. >6 weeks, and as a continuous variable. For analyses of PRO measures, only patients who completed preoperative and 1‐year surveys at both surgeries were included. Models were adjusted for ASA, CCI, BMI, age, sex and smoking status recorded at the time of the second surgery along with any complications from the first stage. To ensure assumptions were met for regression modelling, data were examined for extreme outliers, and independent variables were inspected for multicollinearity using variance inflation factors (VIF). Statistical significance was defined as *p* < 0.05. SPSS (IBM) version 29.0 was used for statistical analysis.

## RESULTS

### Case inclusion and grouping

Among 11,191 primary TKAs within the inclusion dates, 8694 patients had unilateral TKA without a subsequent contralateral procedure and 87 patients received simultaneous BTKAs. A total of 2410 sets of staged BTKA procedures were included. There were 594 cases in Q1 (≤98 days), 618 cases in Q2 (>98 to ≤245 days), 596 cases in Q3 (>245 to ≤571 days), and 602 cases in Q4 (>571 days). The median time between stages was 245 days (mean = 455.65 days). There were 231 (9.6%) subsequent procedures performed within 6 weeks of the first surgery, and 2179 (90.4%) performed greater than 6 weeks after the first stage. There were 1273 patients who completed the KOOS‐JR prior to the first surgery and 706 (47.0%) who completed all KOOS‐JR surveys for both surgeries. There were 1501 patients who completed the VRS‐12 PCS and MCS prior to the first surgery, while 923 (61.5%) and 905 (60.3%) completed all VRS‐12 PCS and MCS surveys for both surgeries, respectively.

### Patient demographics and perioperative variables

The univariable analysis of demographic and surgical variables (at the time of the second surgery) across time quartiles is displayed in Table 1a. Statistically significant differences were observed in age, sex, BMI, CCI scores, and ASA classification (all *p* < 0.001). A greater proportion of patients in Q3 failed to meet MCID for VRS‐12 PCS scores after their first procedure, whereas Q1 had the lowest proportion of patients who failed to meet MCID (*p* < 0.001).

**Table 1a:** Univariable analysis of demographic and surgical variables based on surgical timing: Quartiles.

Preoperative variables	Total (*N* = 2410)	≤98 days (*n* = 594)	>98 to ≤ 245 days (*n* = 618)	>245 to ≤ 571 days (*n *= 596)	>571 days (*n* = 602)	*p*‐Value
Age, years	66.2 ± 8.5	64.9 ± 8.3^a^	67.1 ± 8.5^b^	66.8 ± 8.3^b^	66.2 ± 8.5^a,b^	<0.001
Sex						
Women	1535 (63.7)	320 (53.9)^a^	423 (68.4)^b^	400 (67.1)^b^	392 (65.1)^b^	
Men	875 (36.3)	274 (46.1)^a^	195 (31.6)^b^	196 (32.9)^b^	210 (34.9)^b^	<0.001
BMI, kg/m^2^	33.0 ± 6.7	32.6 ± 6.0^‡^	33.7 ± 7.2^*§^	33.4 ± 6.9^§^	32.3 ± 6.6^‡†^	0.001
CCI						
≤1	323 (13.4)	96 (16.2)	81 (13.1)	66 (11.1)	80 (13.3)	<0.001
2	721 (29.9)	209 (35.2)^a^	150 (24.3)^b^	184 (30.9)^a,b^	178 (29.6)^a,b^	
3	770 (32.0)	191 (32.2)	207 (33.5)	192 (32.2)	180 (29.9)	
4	412 (17.1)	70 (11.8)^a^	113 (18.3)^b^	108 (18.1)^b^	121 (20.1)^b^	
≥5	184 (7.6)	28 (4.7)^a^	67 (10.8)^b^	46 (7.7)^a,b^	43 (7.1)^a,b^	
ASA class						
I–II	1597 (66.3)	419 (70.5)^a^	369 (59.7)^b^	375 (62.9)^b^	434 (72.1)^a^	
III–IV	813 (33.7)	175 (29.5)^a^	249 (40.3)^b^	221 (37.1)^b^	168 (27.9)^a^	<0.001
Smoking status						
Never	1233 (51.2)	321 (54.0)	310 (50.2)	308 (51.7)	294 (48.8)	0.115
Former	1086 (45.1)	245 (41.2)	284 (46.0)	275 (46.1)	282 (46.8)	
Active	91 (3.8)	28 (4.7)	24 (3.9)	13 (2.2)	26 (4.3)	
Any complication after first stage	94 (3.9)	25 (4.2)	18 (2.9)	23 (3.9)	28 (4.7)	0.446
KOOS‐JR	*n* = 706	*n* = 331	*n* = 336	*n* = 317	*n* = 380	
Preoperative: Stage 2	51.2 ± 12.2	52.5 ± 13.5	51.7 ± 12.1	50.0 ± 11.5	50.4 ± 11.4	0.183
Failed to meet MCID: Stage 1	129 (18.3)	24 (13.8)	37 (19.7)	39 (21.8)	29 (17.6)	0.249
VRS‐12 PCS	*n* = 923	*n* = 238	*n* = 237	*n* = 231	*n* = 217	
Preoperative: Stage 2	30.0 ± 8.4	30.4 ± 8.3	29.4 ± 8.2	29.9 ± 8.8	30.3 ± 8.5	0.578
Failed to meet MCID: Stage 1	145 (15.7)	21 (8.8)^a^	32 (13.5)^b^	60 (26.0)^c^	32 (14.7)^b^	<0.001
VRS‐12 MCS	*n* = 905	*n* = 230	*n* = 233	*n* = 226	*n* = 216	
Preoperative: Stage 2	55.3 ± 9.8	55.7 ± 9.5	55.0 ± 10.1	55.4 ± 9.8	54.9 ± 9.7	0.758
Failed to meet MCID: Stage 1	285 (31.5)	75 (32.6)	80 (34.3)	67 (29.6)	63 (29.2)	0.593

*Note*: Groups not sharing a common superscript differ significantly after Bonferroni‐adjusted post hoc comparisons. ‘*’ Significantly different from Q1 after Bonferroni‐adjusted post hoc testing. ‘‡’ Significantly different from Q2 after Bonferroni‐adjusted post hoc testing. ‘†’ Significantly different from Q3 after Bonferroni‐adjusted post hoc testing. ‘§’ Significantly different from Q4 after Bonferroni‐adjusted post hoc testing. Significance level set at *p* ≤ 0.05. Categorical variables displayed as frequency (%). Continuous variables displayed as mean ± SD. Fisher's exact test used for cell frequency counts < 5. Preoperative: Stage 2 = baseline patient‐reported outcome score prior to the second stage procedure. Failed to Meet MCID: Stage 1 = failed to meet MCID on patient‐reported outcome score at 1‐year after the first surgery.

Abbreviations: ASA, American Society of Anesthesiologists; BMI, body mass index; CCI, Charlson comorbidity index; KOOS‐JR, knee injury and osteoarthritis outcome score for joint replacement; MCID, minimal clinically important difference; MCS, mental component score; PCS, physical component score; VRS‐12, veteran RAND 12‐item survey.

The univariable analysis of demographic and surgical variables between procedures performed within a 6‐week timespan compared to those performed greater than 6 weeks apart is shown in Table 1b. The group with stages greater than 6 weeks apart were older (*p* < 0.001), had a higher BMI (*p* < 0.001), higher CCI (*p* < 0.001), and ASA classification (*p* < 0.001) compared to the less than 6 weeks group. There was a greater proportion of patients in the less than 6 weeks group who experienced a complication after the first procedure (6.5% vs. 3.6%; *p* = 0.032). A greater proportion of patients with stages more than 6 weeks apart failed to meet MCID for VRS‐12 PCS scores after their first procedure (*p* = 0.043).

**Table 1b:** Univariable analysis of demographic and surgical variables based on surgical timing: Binary.

Preoperative variables	Total (*N* = 2410)	≤6 weeks (*n* = 231)	>6 weeks (*n* = 2179)	*p*‐Value
Age, years	66.2 ± 8.5	64.3 ± 8.0	66.4 ± 8.5	<0.001
Sex				
Women	1535 (63.7)	121 (52.4)	1414 (64.9)	<0.001
Men	875 (36.3)	110 (47.6)	765 (35.1)	
BMI, kg/m^2^	33.0 ± 6.7	31.3 ± 4.6	33.2 ± 6.9	<0.001
CCI				
≤1	323 (13.4)	50 (21.6)	273 (12.5)	
2	721 (29.9)	77 (33.3)	644 (29.6)	
3	770 (32.0)	70 (30.3)	700 (32.1)	
4	412 (17.1)	26 (11.3)	386 (17.7)	
≥5	184 (7.6)	8 (3.5)	176 (8.1)	<0.001
ASA class				
I–II	1597 (66.3)	179 (77.5)	1418 (65.1)	<0.001
III–IV	813 (33.7)	52 (22.5)	761 (34.9)	
Smoking status				
Never	1233 (51.2)	114 (49.4)	1119 (51.4)	
Former	1086 (45.1)	106 (45.9)	980 (45.0)	
Active	91 (3.8)	11 (4.8)	80 (3.7)	0.650
Any complication: Stage 1	94 (3.9)	15 (6.5)	79 (3.6)	0.032
KOOS‐JR	*n* = 706	*n* = 62	*n* = 644	
Preoperative: Stage 2	51.2 ± 12.2	51.4 ± 13.8	51.2 ± 12.0	0.350
Failed to meet MCID: Stage 1	129 (18.3)	12 (19.4)	117 (18.2)	0.817
VRS‐12 PCS	*n* = 923	*n* = 94	*n* = 829	
Preoperative: Stage 2	30.0 ± 8.4	30.4 ± 8.2	30.0 ± 8.5	0.955
Failed to meet MCID: Stage 1	145 (15.7)	8 (8.5)	137 (16.5)	0.043
VRS‐12 MCS	*n* = 905	*n* = 92	*n* = 813	
Preoperative: Stage 2	55.3 ± 9.8	54.7 ± 10.4	55.3 ± 9.7	0.613
Failed to meet MCID: Stage 1	285 (31.5)	34 (37.0)	251 (30.9)	0.234

*Note*: Significance level set at *p* ≤ 0.05. Categorical variables displayed as frequency (%). Continuous variables displayed as mean ± SD. Fisher's exact test used for cell frequency counts < 5. Preoperative: Stage 2 = baseline patient‐reported outcome score prior to the second stage procedure. Failed to Meet MCID: Stage 1 = failed to meet MCID on patient‐reported outcome score at 1‐year after the first surgery.

Abbreviations: ASA, American Society of Anesthesiologists; BMI, body mass index; CCI, Charlson comorbidity index; KOOS‐JR, knee injury and osteoarthritis outcome score for joint replacement; MCID, minimal clinically important difference; MCS, mental component score; PCS, physical component score; VRS‐12, veteran RAND 12‐item survey.

### 90‐day complication rates after second stage

The frequency of 90‐day complications between time quartiles is shown in Table 2a. There were no significant differences in overall 90‐day complications (*p* = 0.472), ED visits (*p* = 0.849), hospital readmissions (*p *= 0.734), return to operating room (RTOR) (*p* = 0.656) or PJI (*p* = 0.777) across quartiles. After the second stage, 1‐year postoperative VRS‐12 PCS scores were highest in the Q1 group (*p* < 0.001), while MCS scores were higher in Q1 compared to Q3 (*p* = 0.027). A greater proportion of patients in Q3 failed to meet MCID on PCS scores compared to all other groups (*p* < 0.001).

**Table 2a:** 90‐day complications and 1‐year postoperative PROs following the second stage: Quartiles.

Variable	Total (*N* = 2410)	≤98 days (*n* = 594)	>98 to ≤245 days (*n* = 618)	>245 to ≤571 days (*n* = 596)	>571 days (*n* = 602)	*p*‐Value
Any complication	114 (4.7)	28 (4.7)	31 (5.0)	33 (5.5)	22 (3.7)	0.472
ED visit	104 (4.3)	28 (4.7)	25 (4.0)	23 (3.9)	28 (4.7)	0.849
Readmission	63 (2.6)	16 (2.7)	14 (2.3)	19 (3.2)	14 (2.3)	0.734
Reoperation	33 (1.4)	6 (1.0)	11 (1.8)	9 (1.5)	7 (1.2)	0.656
PJI	18 (0.7)	3 (0.5)	5 (0.8)	6 (1.0)	4 (0.7)	0.777
VTE	3 (0.1)	1 (0.2)	0 (0.0)	0 (0.0)	2 (0.3)	0.296
Dislocations	0 (0.0)	0 (0.0)	0 (0.0)	0 (0.0)	0 (0.0)	—
Mechanical failure	0 (0.0)	0 (0.0)	0 (0.0)	0 (0.0)	0 (0.0)	—
Periprosthetic fracture	3 (0.1)	0 (0.0)	1 (0.2)	2 (0.3)	0 (0.0)	0.296
Tendon rupture	7 (0.3)	3 (0.5)	0 (0.0)	0 (0.0)	4 (0.7)	0.060
SSI	4 (0.2)	1 (0.2)	0 (0.0)	3 (0.5)	0 (0.0)	0.106
Wound dehiscence	7 (0.3)	3 (0.5)	2 (0.3)	0 (0.0)	2 (0.3)	0.433
Mortality	2 (0.1)	1 (0.2)	1 (0.2)	0 (0.0)	0 (0.0)	0.577
KOOS‐JR	*n* = 706	*n* = 174	*n* = 188	*n* = 179	*n* = 165	
One‐year postoperative	78.3 ± 13.7	80.7 ± 12.3	77.7 ± 13.3	76.4 ± 14.4	78.5 ± 14.2	0.054
Failed to meet MCID	181 (25.6)	48 (27.6)	53 (28.2)	43 (24.0)	37 (22.4)	0.546
VRS‐12 PCS	*n* = 923	*n* = 238	*n* = 237	*n* = 231	*n* = 217	
One‐year postoperative	45.9 ± 8.8	48.3 ± 7.7^a^	45.7 ± 8.7^b^	43.8 ± 9.6^b^	45.7 ± 8.7^b^	<0.001
Failed to meet MCID	193 (20.9)	35 (14.7)^a^	49 (20.7)^a^	69 (29.9)^b^	40 (18.4)^a^	<0.001
VRS‐12 MCS	*n* = 905	*n* = 230	*n* = 233	*n* = 226	*n* = 216	
One‐year postoperative	56.5 ± 8.1	57.7 ± 7.2^a^	55.9 ± 8.7^a^	55.7 ± 8.7^b^	56.5 ± 7.6^a^	0.027
Failed to meet MCID	280 (30.9)	80 (34.8)	69 (29.6)	64 (28.3)	67 (31.0)	0.474

*Note*: Groups not sharing a common superscript differ significantly after Bonferroni‐adjusted post hoc comparisons. Significance level set at *p* ≤ 0.05. Categorical variables displayed as frequency (%). Continuous variables displayed as mean ± SD. Fisher's exact test used for cell frequency counts < 5. One‐year postoperative = mean patient‐reported outcome score at 1‐year after the second surgery, failed to meet MCID = failed to meet MCID on 1‐year patient reported outcome score at 1‐year after the second surgery.

Abbreviations: ED, emergency department; KOOS‐JR, knee injury and osteoarthritis outcome score for joint replacement; MCID, minimal clinically important difference; MCS, mental component score; PCS, physical component score; PJI, periprosthetic joint infection; PRO, patient‐reported outcome; RTOR, return to operating room; SSI, surgical site infection; VRS‐12, veteran RAND 12‐item survey; VTE, venous thromboembolism.

The frequencies of 90‐day complications between stages performed within 6 weeks and greater than 6 weeks apart are presented in Table 2b. There were no significant differences in overall 90‐day complications (*p* = 0.981), ED visits (*p* = 0.177), hospital readmissions (*p* = 0.199), RTOR (*p* = 0.999), or PJI (*p* = 0.407). After the second stage, 1‐year VRS‐12 PCS scores were greater in the less than 6 weeks cohort (*p* = 0.020), with a smaller proportion of patients failing to meet MCID on PCS scores (*p* = 0.040).

**Table 2b:** 90‐day complications and 1‐year postoperative PROs following the second stage: Binary.

Variable	Total (*N* = 2410)	≤6 weeks (*n* = 231)	>6 weeks (*n* = 2179)	*p*‐Value
Any complication	114 (4.7)	11 (4.8)	103 (4.7)	0.981
ED visit	104 (4.3)	6 (2.6)	98 (4.5)	0.177
Readmission	63 (2.6)	9 (3.9)	54 (2.5)	0.199
Reoperation	33 (1.4)	3 (1.3)	30 (1.4)	0.999
PJI	18 (0.7)	0 (0.0)	18 (0.8)	0.407
VTE	3 (0.1)	0 (0.0)	3 (0.1)	0.999
Dislocations	0 (0.0)	0 (0.0)	0 (0.0)	—
Mechanical failure	0 (0.0)	0 (0.0)	0 (0.0)	—
Periprosthetic fracture	3 (0.1)	0 (0.0)	3 (0.1)	0.999
Tendon rupture	7 (0.3)	2 (0.9)	5 (0.2)	0.139
SSI	4 (0.2)	1 (0.4)	3 (0.1)	0.332
Wound dehiscence	7 (0.3)	2 (0.9)	5 (0.2)	0.139
Mortality	2 (0.1)	0 (0.0)	2 (0.1)	0.999
KOOS‐JR	*n* = 706	*n* = 62	*n* = 644	
One‐year postoperative	78.3 ± 13.6	78.5 ± 11.8	78.3 ± 13.8	0.967
Failed to meet MCID	181 (25.6)	21 (33.9)	160 (24.8)	0.120
VRS‐12 PCS	*n* = 923	*n* = 94	*n* = 829	
One‐year postoperative	45.9 ± 8.8	47.7 ± 8.0	45.7 ± 8.9	0.020
Failed to meet MCID	193 (20.9)	12 (12.8)	181 (21.8)	0.040
VRS‐12 MCS	*n* = 905	*n* = 92	*n* = 813	
One‐year postoperative	56.5 ± 8.1	56.9 ± 8.5	56.4 ± 8.1	0.612
Failed to meet MCID	280 (30.9)	31 (33.7)	249 (30.6)	0.546

*Note*: Significance level set at *p* ≤ 0.05. Categorical variables displayed as frequency (%). Continuous variables displayed as mean ± SD. Fisher's exact test used for cell frequency counts < 5. One‐year postoperative = mean patient‐reported outcome score at 1‐year after the second surgery, Failed to meet MCID, failed to meet MCID on 1‐year patient reported outcome score at 1‐year after the second surgery.

Abbreviations: ED, emergency department; KOOS‐JR, knee injury and osteoarthritis outcome score for joint replacement; MCID, minimal clinically important difference; MCS, mental component score; PCS, physical component score; PJI, periprosthetic joint infection; PRO, patient‐reported outcome; RTOR, return to operating room; SSI, surgical site infection; VRS‐12, veteran RAND 12‐item survey; VTE, venous thromboembolism.

### Complications

Multivariable logistic regression evaluating predictors of 90‐day complications following the second procedure is represented in Table 3. Although several univariable differences in complications were observed across timing groups, these associations were no longer significant after multivariable adjustment. Whether assessed as quartiles (*p* = 0.407), ≤6 versus >6 weeks (*p* = 0.895), or as a continuous variable in 30‐day intervals (*p* = 0.892), timing was not independently associated with complication risk. The strongest predictor across all models was having experienced any complication after the first surgery (odds ratio [OR] = 4.01, 95% confidence interval [CI] = 2.21−7.26; *p* < 0.001). An ASA classification of III–IV (OR = 1.60, 95% CI = 1.05−2.43; *p* = 0.028) was also consistently associated with an increased risk of any complication after the second stage.

**Table 3:** Multivariable logistic regressions predicting 90‐day complications after second stage.

	Variable	OR (95% CI)	*p*‐Value
Quartile based	Time: Quartiles	1.08 (0.90–1.28)	0.407
	Prior complication after first stage	4.05 (2.24–7.33)	<0.001
	ASA III–IV	1.59 (1.05–2.41)	0.030
≤6 vs. >6 weeks	Time: Binary	1.05 (0.54–2.01)	0.895
	Prior complication after first stage	4.00 (2.21–7.26)	<0.001
	ASA III–IV	1.60 (1.05–2.42)	0.028
Continuous (per 30 day)	Time: Continuous scale	1.00 (0.99–1.01)	0.892
	Prior complication after first stage	4.01 (2.21–7.26)	<0.001
	ASA III–IV	1.60 (1.05–2.43)	0.028

*Note*: Included in the model as covariates: ASA, CCI, BMI, age, sex, and smoking status at the second surgery. Reference categories for categorical variables: Q1, no complication, non‐smoker, ASA I–II, female.

Abbreviations: ASA, American Society of Anesthesiologists; BMI, body mass index; CCI, Charlson comorbidity index; CI, confidence interval; OR, odds ratio.

### Failure to meet MCID at 1‐year

Multivariable logistic regression evaluating predictors of failure to meet MCID on KOOS‐JR (+16 points), VRS‐12 PCS (+5 points) and VRS‐12 MCS (+5 points) at 1 year are displayed in Table 4a, Table 4b, and Table 4c, respectively. Although several univariable differences in PROs and MCID achievement were observed across timing groups, these associations were no longer significant after multivariable adjustment. Across all models, timing was not independently associated with failure to meet MCID on any PROs. Failure to achieve MCID 1‐year after the first surgery was the strongest predictor of subsequent failure on KOOS‐JR (OR = 26.34, 95% CI = 15.15−45.78; *p* < 0.001), VRS‐12 PCS (OR = 31.67, 95% CI = 18.22−55.06; *p* < 0.001), and VRS‐12 MCS (OR = 8.69, 95% CI = 5.78−13.06; *p* < 0.001). Higher average preoperative PRO scores at the second stage were also associated with an increased risk for KOOS‐JR (OR = 1.11, 95% CI = 1.08−1.16; *p* < 0.001), VRS‐12 PCS (OR = 1.15, 95% CI = 1.12−1.20; *p* < 0.001), and VRS‐12 MCS (OR = 1.19, 95% CI = 1.16−1.22; *p* < 0.001). In addition, active smoking status was associated with failure to meet MCID for VRS‐12 PCS (OR = 1.62, 95% CI = 1.08−2.43; *p* = 0.019), whereas an ASA classification III–IV was associated with failure on VRS‐12 MCS (OR = 2.29, 95% CI = 1.38−3.79; *p* = 0.001).

**Table 4a:** Multivariable logistic regressions predicting failure to achieve MCID on KOOS‐JR at 1‐year after second stage.

	Variable	OR (95% CI)	*p*‐Value
Quartile based	Time: Quartiles	1.01 (0.99–1.03)	0.158
	Failed to achieve MCID after first stage	26.52 (15.23–46.17)	<0.001
	Prior complication after first stage	1.40 (0.44–4.46)	0.575
	Preoperative KOOS‐JR	1.12 (1.09–1.15)	<0.001
≤6 vs. >6 weeks	Time: Binary	1.72 (0.82–3.57)	0.148
	Failed to achieve MCID after first stage	25.65 (14.82–44.39)	<0.001
	Prior complication after first stage	1.56 (0.48–5.05)	0.458
	Preoperative KOOS‐JR	1.12 (1.08–1.15)	<0.001
Continuous (per 30 day)	Time: Continuous scale	1.15 (0.94–1.41)	0.171
	Failed to achieve MCID after first stage	26.34 (15.15–45.78)	<0.001
	Prior complication after first stage	1.48 (0.46–4.71)	0.512
	Preoperative KOOS‐JR	1.11 (1.08–1.16)	<0.001

*Note*: Included in the model as covariates: ASA, CCI, BMI, age, sex, and smoking status at the second surgery. Reference categories for categorical variables: Q1 and ≤6 weeks, achieved MCID after index, no complication, non‐smoker, ASA I–II, female.

Abbreviations: ASA, American Society of Anesthesiologists; BMI, body mass index; CCI, Charlson comorbidity index; CI, confidence interval; KOOS‐JR, knee injury and osteoarthritis outcome score for joint replacement; MCID, minimal clinically important difference; OR, odds ratio.

**Table 4b:** Multivariable logistic regressions predicting failure to achieve MCID on VRS‐12 PCS at 1‐year after second stage.

	Variable	OR (95% CI)	*p*‐Value
Quartile based	Time: Quartiles	1.09 (0.90–1.35)	0.349
	Failed to achieve MCID after first stage	30.66 (17.61–53.37)	<0.001
	Prior complication after first stage	2.02 (0.50–8.14)	0.323
	Preoperative VRS‐12 PCS	1.16 (1.12–1.20)	<0.001
	Smoking status	1.62 (1.08–2.43)	0.019
≤6 vs. >6 weeks	Time: Binary	1.60 (0.64–3.97)	0.312
	Failed to achieve MCID after first stage	31.27 (18.00–54.31)	<0.001
	Prior complication after first stage	1.93 (0.49–7.62)	0.347
	Preoperative VRS‐12 PCS	1.16 (1.12–1.20)	<0.001
	Smoking status	1.62 (1.08–2.43)	0.019
Continuous (per 30 day)	Time: Continuous scale	0.99 (0.98–1.01)	0.891
	Failed to achieve MCID after first stage	31.67 (18.22–55.06)	<0.001
	Prior complication after first stage	2.01 (0.50–8.11)	0.328
	Preoperative VRS‐12 PCS	1.15 (1.12–1.20)	<0.001
	Smoking status	1.62 (1.08–2.43)	0.019

*Note*: Included in the model as covariates: ASA, CCI, BMI, age, sex and smoking status at the second surgery. Reference categories for categorical variables: Q1 and ≤6 weeks, achieved MCID after index, no complication, non‐smoker, ASA I–II, female.

Abbreviations: ASA, American Society of Anesthesiologists; BMI, body mass index; CCI, Charlson comorbidity index; CI, confidence interval; MCID, minimal clinically important difference; OR: odds ratio; VRS‐12 PCS, veterans RAND 12‐item survey physical component score.

**Table 4c:** Multivariable logistic regressions predicting failure to achieve MCID on VRS‐12 MCS at 1‐year after second stage.

	Variable	OR (95% CI)	*p*‐Value
Quartile based	Time: Quartiles	1.02 (0.86–1.23)	0.755
	Failed to achieve MCID after first stage	8.46 (5.63–12.70)	<0.001
	Prior complication after first stage	3.86 (1.11–13.51)	0.034
	Preoperative VRS‐12 MCS	1.19 (1.15–1.22)	<0.001
	ASA III–IV	2.21 (1.33–3.65)	0.002
≤6 vs. >6 weeks	Time: Binary	1.03 (0.52–2.04)	0.924
	Failed to achieve MCID after first stage	8.50 (5.67–12.8)	<0.001
	Prior complication after first stage	3.85 (1.11–14.3)	0.033
	Preoperative VRS‐12 MCS	1.18 (1.15–1.22)	<0.001
	ASA III–IV	2.23 (1.34–3.68)	0.002
Continuous (per 30 day)	Time: Continuous scale	0.99 (0.98–1.02)	0.096
	Failed to achieve MCID after first stage	8.69 (5.78–13.06)	<0.001
	Prior complication after first stage	4.21 (1.20–14.93)	0.025
	Preoperative VRS‐12 MCS	1.19 (1.16–1.22)	<0.001
	ASA III–IV	2.29 (1.38–3.79)	0.001

*Note*: Included in the model as covariates: ASA, CCI, BMI, age, sex and smoking status at the second surgery. Reference categories for categorical variables: Q1 and ≤6 weeks, achieved MCID after index, no complication, non‐smoker, ASA I–II, female.

Abbreviations: ASA, American Society of Anesthesiologists; BMI, body mass index; CCI, Charlson comorbidity index; CI, confidence interval; MCID, minimal clinically important difference; OR: odds ratio; VRS‐12 PCS, veterans RAND 12‐item survey physical component score.

## DISCUSSION

This study evaluated whether inter‐stage timing between staged BTKA procedures influences 90‐day complications or 1‐year functional outcomes. Across all models, inter‐stage timing was not independently associated with postoperative complications or failure to achieve MCID after adjustment for patient‐level factors. Similarly, patients undergoing the second stage within 6 weeks of their first surgery did not demonstrate increased rates of adverse outcomes or inferior functional recovery. Instead, postoperative outcomes appeared to be driven primarily by prior surgical outcomes, ASA classification, and baseline PRO scores.

Prior literature on staging intervals has been mixed. Agarwal et al. reported higher complication and revision rates with intervals under 6 weeks, although their analysis lacked key covariates such as ASA classification and prior complications after the first stage [1]. Richardson et al. similarly reported higher risk with shorter intervals but did not account for prior surgical response [24]. In contrast, several other studies found no clear association between interval length and postoperative outcomes, although many were limited by smaller sample sizes or limited adjustment for patient‐level factors [4, 7, 14, 21]. By modelling timing both categorically and continuously while accounting for patient‐level factors and prior surgical outcomes, the present study suggests that interval length alone may exert less influence on postoperative outcomes than patient‐level factors and prior surgical response. Univariable analyses demonstrated differences in VRS‐12 PCS outcomes across timing quartiles, however, these associations were no longer significant after multivariable adjustment. Patients undergoing earlier second stage surgery differed in several baseline characteristics including age, ASA classification, comorbidity burden, and postoperative recovery after the first stage. After adjustment for these factors, inter‐stage timing was not independently associated with PRO outcomes, suggesting that the observed univariable differences were more likely attributable to underlying patient characteristics. Notably, prior failure to achieve MCID after the first surgery demonstrated substantially stronger associations with outcomes after the second stage than timing alone.

The strongest and most consistent finding of this study was the predictive value of prior surgical response, which aligns with reports by Grace et al. [8] and Schwarzkopf et al. [28]. Patients who experienced complications after the first stage were substantially more likely to experience complications after the second stage, while patients who failed to achieve MCID after the first stage demonstrated markedly increased odds of failing to achieve MCID again following the second surgery. Together, these findings suggest that prior surgical response and postoperative recovery after the first procedure may be more informative than inter‐stage timing alone when estimating subsequent surgical risk and functional recovery. These associations may also reflect broader patient‐level recovery characteristics including systemic functional status, psychosocial factors, generalised musculoskeletal limitations, comorbidity burden, or patient‐specific recovery trajectory rather than isolated knee pathology alone. In addition, ASA classification independently predicted complications, consistent with literature linking ASA III–IV to higher infection, dislocation, thromboembolic, and mortality following arthroplasty procedures [10, 15, 17, 22, 25, 27, 32]. We also found that patients with higher preoperative PROs were less likely to achieve MCID, aligning with prior studies [31, 34]. This difference likely reflects a ceiling effect, as patients with higher baseline scores are less likely to meet MCID thresholds and does not actually preclude patients with higher preoperative scores from successful outcomes.

Clinically, these findings support individualised staging decisions and emphasise the importance of assessing recovery and surgical response on an individual basis rather than relying solely on interval timing when planning the second stage of BTKA. Patients who experience complications or fail to achieve MCID after the first stage may represent higher risk subgroups for subsequent adverse outcomes and may benefit from additional optimisation and expectation management prior to proceeding with the second surgery, irrespective of timing interval. Conversely, patients who demonstrate an uncomplicated recovery and meaningful functional improvement after the first procedure may be better suited to tailor timing of the second‐stage surgery based on symptom severity and logistical considerations through a shared decision‐making process. Although the relatively small subgroup of patients who underwent the second surgery within 6 weeks of the first procedure limits definitive conclusions regarding shorter staging intervals, the absence of an observed increase in adverse outcomes suggests that earlier staging strategies may be reasonable in carefully selected patients who recover appropriately after the first procedure. Future studies with larger early‐staged cohorts are warranted to further evaluate the relationship between shorter staging intervals and postoperative outcomes. Accordingly, surgical planning should incorporate patient recovery, comorbidity burden, rehabilitation progress and patient goals rather than relying solely on a fixed temporal threshold.

### Limitations

This study has several limitations inherent to its retrospective design. Surgical timing was not randomised or controlled and likely reflects both clinical and nonclinical factors such as symptom severity, patient motivation, health status, recovery from the first surgery, logistical constraints, surgeon discretion and scheduling availability. Patients undergoing earlier second‐stage surgery may have also represented a healthier subgroup with more favourable recovery after the first stage, introducing the potential for confounding by indication and selection bias that may not have been fully accounted for despite multivariable adjustment. In addition, some cases may have been delayed due to prior poor outcomes, while others may have not been necessary until minor pathology progressed to require surgery. The subgroup of patients who underwent the second stage within 6 weeks was relatively small, which may have limited statistical power to detect smaller but meaningful differences between early and delayed staging groups. Therefore, the absence of an observed association should not be interpreted as definitive evidence of equivalence between staging approaches. Several relevant variables, including implant selection, surgical technique or approach, anaesthesia modality, rehabilitation protocols and temporal changes in perioperative management, were not included in this study. The complication outcome evaluated in the present study pooled heterogenous postoperative events, as individual endpoints such as PJI, DVT and MI events were sparse and would have likely been underpowered. Although PRO response rates were relatively strong, nonresponse bias remains possible, particularly among patients with limited access, lower health literacy, or non‐English speakers who may have been less likely to complete surveys and therefore may have been inadvertently underrepresented [19]. Additionally, PRO analyses were restricted to complete‐case patients with available survey data from both the first and second stage, introducing the potential for selection bias and limited generalisability. Multiple multivariable models evaluating distinct timing constructs and outcomes were performed without a formal multiplicity adjustment, which may increase the risk of type I error. Finally, this was a single‐institution study at a high‐volume centre, which may limit generalisability to other practice settings.

## CONCLUSION

Timing between staged BTKA procedures was not independently associated with 90‐day complications or failure to achieve meaningful functional improvement after adjustment for patient‐level factors. Instead, prior surgical response, recovery following the first stage, and higher ASA classification demonstrated stronger associations with postoperative outcomes following the second stage procedure. These findings suggest that patient recovery and response after the first stage may be more informative for surgical planning than interval duration alone. Individualised surgical planning guided by patient‐level risk, postoperative recovery, and shared decision‐making with targeted preoperative optimisation and expectation management may be more clinically meaningful than reliance on fixed interval thresholds.

## AUTHOR CONTRIBUTIONS

**Jake M. Laverdiere**: Conceptualisation; methodology; data collection and analysis; writing—original draft preparation; writing—review and editing. **Ekrem M. Ayhan**: Methodology; data collection and analysis; writing—original draft preparation; writing—review and editing. **Thomas P. Giannasca**: Methodology; data collection and analysis; writing—original draft preparation; writing—review and editing. **Jordan A. Bauer**: Methodology; data collection and analysis; writing—original draft preparation; writing—review and editing. **Baseel M. Hamideh**: Data collection and analysis; writing—original draft preparation; writing—review and editing. **Martinus Megalla**: writing—review and editing. **Matthew J. Grosso**: Conceptualisation; writing—review and editing; supervision. All authors have read and approved the final manuscript.

## FUNDING INFORMATION

The authors have no funding to report.

## CONFLICT OF INTEREST STATEMENT

The authors declare no conflicts of interest.

## ETHICS STATEMENT

This study was performed in line with the principles of the Declaration of Helsinki. Approval was granted by the Ethics Committee of Trinity Health of New England under IRB SFH‐25‐12 on 2/26/25.

## Data Availability

The data that support the findings of this study are available on request from the corresponding author. The data are not publicly available due to privacy or ethical restrictions.
